# Enhanced Orai1-mediated store-operated Ca^2+^ channel/calpain signaling contributes to high glucose-induced podocyte injury

**DOI:** 10.1016/j.jbc.2022.101990

**Published:** 2022-04-29

**Authors:** Yu Tao, Sarika Chaudhari, Parisa Yazdizadeh Shotorbani, Yanfeng Ding, Zhenglan Chen, Ramesh Kasetti, Gulab Zode, Rong Ma

**Affiliations:** 1 Department of Physiology and Anatomy, University of North Texas Health Science Center, Fort Worth, Texas, USA; 2 The North Texas Eye Research Institute and Department of Pharmacology and Neuroscience, University of North Texas Health Science Center, Fort Worth, Texas, USA

**Keywords:** podocyte, diabetic nephropathy, calpain, CRISPR-Cas9, calcium release-activated calcium channel protein 1 (ORAI1), Ang II, angiotensin II, BTP, bis(trifluoromethyl) pyrazole, Cas9, CRISPR-associated protein 9, CT, control, DN, diabetic nephropathy, ER, endoplasmic reticulum, FBS, fetal bovine serum, HG, high glucose, HPC, human podocyte, HS, high osmolality, LV, lentivirus, NG, normal glucose, RT, room temperature, SOC, store-operated Ca^2+^ channel, SOCE, store-operated Ca^2+^ entry, STIM1, stromal interaction molecule 1, TG, thapsigargin, TRPC, transient receptor potential canonical

## Abstract

Podocyte injury induced by hyperglycemia is the main cause of kidney dysfunction in diabetic nephropathy. However, the underlying mechanism is unclear. Store-operated Ca^2+^ entry (SOCE) regulates a diversity of cellular processes in a variety of cell types. Calpain, a Ca^2+^-dependent cysteine protease, was recently shown to be involved in podocyte injury. In the present study, we sought to determine whether increased SOCE contributed to high glucose (HG)–induced podocyte injury through activation of the calpain pathway. In cultured human podocytes, whole-cell patch clamp indicated the presence of functional store-operated Ca^2+^ channels, which are composed of Orai1 proteins and mediate SOCE. Western blots showed that HG treatment increased the protein abundance of Orai1 in a dose-dependent manner. Consistently, calcium imaging experiments revealed that SOCE was significantly enhanced in podocytes following HG treatment. Furthermore, HG treatment caused overt podocyte F-actin disorganization as well as a significant decrease in nephrin protein abundance, both of which are indications of podocyte injury. These podocyte injury responses were significantly blunted by both pharmacological inhibition of Orai1 using the small molecule inhibitor BTP2 or by genetic deletion of *Orai1* using CRISPR-Cas9 lentivirus. Moreover, activation of SOCE by thapsigargin, an inhibitor of Ca^2+^ pump on the endoplasmic/sarcoplasmic reticulum membrane, significantly increased the activity of calpain, which was inhibited by BTP2. Finally, the calpain-1/calpain-2 inhibitor calpeptin significantly blunted the nephrin protein reduction induced by HG treatment. Taken together, our results suggest that enhanced signaling *via* an Orai1/SOCE/Calpain axis contributes to HG-induced podocyte injury.

Podocytes are terminally differentiated glomerular visceral epithelial cells, which constitute the outer layer of the glomerular filtration barrier. Adjacent podocyte foot processes are bridged by the porous slit diaphragm that forms the final barrier to prevent albumin leakage (1). Accumulating evidence suggests that podocyte injury is the key contributing factor in the pathogenesis of most inherited and acquired glomerular diseases, such as diabetic nephropathy (DN). During the past decade, many studies have demonstrated that diabetes-induced podocyte injury is the primary cause leading to proteinuria and one of the major factors contributing to the occurrence and progression of DN (2, 3). The DN-associated podocyte injury includes foot process effacement, cell detachment, and slit diaphragm disruption, which are further manifested by the podocyte cytoskeleton disorganization and podocyte phenotype marker protein loss. While the etiology of podocyte injury is likely multifactorial, hyperglycemia is the major pathogenic factor to drive podocytes injury in DN (4). However, the molecular mechanisms underlying the hyperglycemia-induced podocyte injury are not known with certainty.

Podocyte structural integrity is dependent on intracellular Ca^2+^ homeostasis. In this regard, the importance of transient receptor potential canonical (TRPC) 5 and TRPC6 channels in maintaining podocyte function and structure has been widely studied (5, 6, 7, 8, 9, 10). Although a recent study by Kim *et al.* reported a contribution of store-operated calcium channel (SOC) to podocyte injury (11), the function of the channel in podocytes and the downstream mechanism mediating the channel function is, at a large extent, not known. SOC is activated upon depletion of the endoplasmic reticulum (ER) Ca^2+^. The plasma membrane–localized Orai1 is the pore-forming unit of the classical SOC (*i.e.*, Ca^2+^ release–activated Ca^2+^ channel). In addition, stromal interaction molecule 1 (STIM1), a transmembrane protein located on ER can sense the ER luminal calcium concentration. Once ER calcium is depleted, STIM1s diffusely distributed on the ER membrane are clustered and relocated to the ER–plasma membrane junctions where they interact with Orai1 channel proteins. The Orai1–STIM1 interactions turn on the Orai1 channels and allow Ca^2+^ to enter the cells (12, 13, 14, 15, 16, 17, 18, 19). This Orai1-mediated Ca^2+^ entry, termed store-operated Ca^2+^ entry (SOCE), has been known to be essential for multiple cellular processes (20). Recently, emerging evidence suggests that the function of SOCE is cell-context and cell-type dependent. For instance, in the proximal tubular epithelial cells Mai *et al.* reported that blockade of SOCE inhibited TGF-β1-stimulated fibronectin protein expression (21). On the contrary, in glomerular mesangial cells, suppression of SOCE increased fibronectin protein abundance (21, 22). Currently, the role of SOCE in podocyte biology and physiology, particularly podocyte injury in the settings of diabetes remains unclear. Calpains belong to the family of Ca^2+^-activated cysteine proteases, which are mainly activated by increased intracellular Ca^2+^ (23). Accumulating evidence suggests that calpain overactivation can induce podocyte injury. However, whether and how calpain contributes to DN-associated podocyte injury has not been studied. In the present study, we aimed to determine that high glucose (HG) treatment enhanced SOCE, and this enhancement resulted in podocyte injury by upregulating calpain activity.

## Results

### SOC in human podocytes

**Figure 1:**
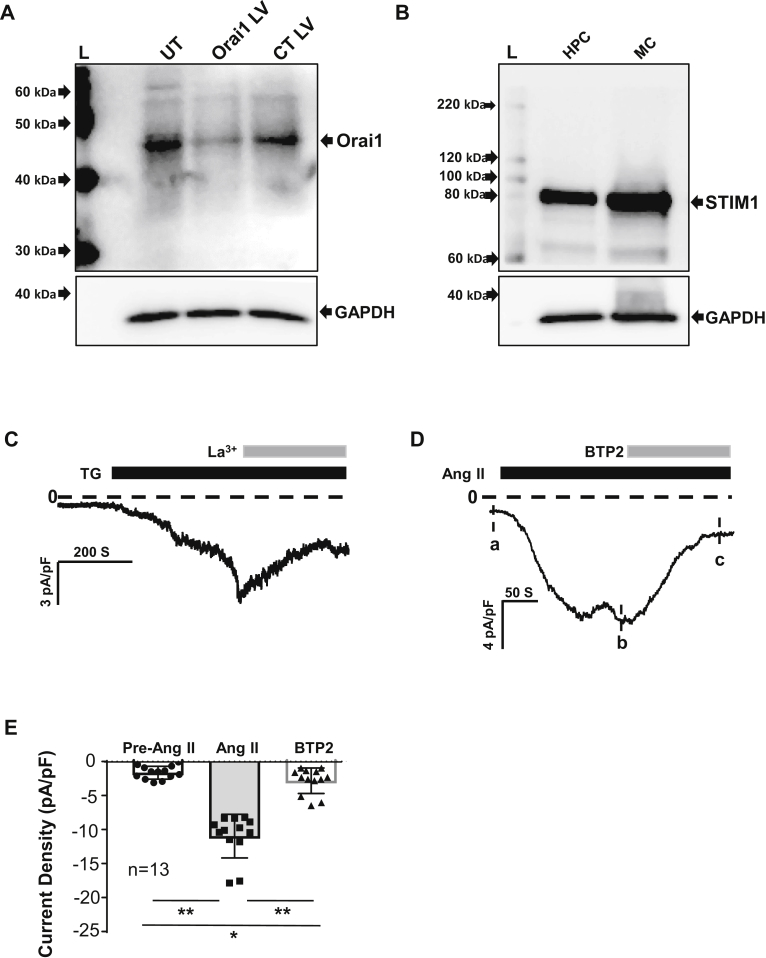
**SOC in cultured HPCs.***A*, Western blots showing expression of Orai1 (A) and STIM1 (B) proteins in HPCs. *A*, the blot is the representative of six independent experiments. Fully differentiated podocytes were infected with CRISPR-Cas9 Orai1 lentivirus (Orai1 LV) or CRISPR-Cas9 control lentivirus (CT LV) or without virus infection (UT). *B*, the lysates from glomerular mesangial cells (MC) were used as the positive control for HPCs. “L”: protein ladder. GAPDH served as the loading control. *C*–*E*, whole cell current measurements obtained by a Gap-free protocol in HPCs stimulated with 2 μM TG (*C*) and 1 μM Ang II (*D* and *E*) at a holding potential of -80 mV. The *dashed lines* indicate zero currents. Application of TG, Ang II, La^3+^, and BTP2 were indicated by *solid lines* on top of the current traces. *D*, the *vertical dashed lines* indicate the place selected for analyzing the currents before administration of Ang II (a), the peak current after Ang II treatment (b), and the current after application of BTP2 (c). *E*, summary data from the experiments presented in (*D*). The currents of pre-Ang II, Ang II, and BTP2 were indicated by a, b, and c in panel (*D*), respectively. ∗ denotes *p* < 0.05; ∗∗ denotes *p* < 0.01. “n”: the number of cells analyzed. Ang II, angiotensin II; BTP, bis(trifluoromethyl) pyrazole; Cas9, CRISPR-associated protein 9; HPC, human podocyte; SOC, store-operated Ca2+ channel; STIM1, stromal interaction molecule 1; TG, thapsigargin.

SOC is an essential Ca^2+^ entry mechanism in both excitable and nonexcitable cells (20). STIM1 and Orai1 are two key components in this channel pathway (12, 13, 14, 15, 16, 17). To verify the existence of SOC in podocytes, we performed Western blot and showed that both Orai1 and STIM1 proteins were present in human podocytes (HPCs) (Fig. 1, *A* and *B*). CRISPR–CRISPR-associated protein 9 (Cas9)/Orai1 lentivirus (Fig. 1*A*, Orai LV) but not CRISPR-Cas9/control LV (Fig. 1*A*, CT LV) deleted the Orai1 antibody-detected band, validating the presence of Orai1 protein in podocytes. Also, the STIM1 antibody-detected band in podocytes was at the same size as that in glomerular mesangial cells (Fig. 1*B*), a cell type known to be rich in STIM1 protein (22, 24, 25), confirming the existence of STIM1 protein in podocytes.

To examine if the Orai1 channel is functional in podocytes, we carried out electrophysiology experiments and measured the channel currents in response to the channel activators and blockers. Because SOC is more conductive to monovalent cations over divalent cations (26, 27, 28, 29), we measured Na^+^ currents using the mode of the whole-cell patch. As shown in Figure 1*C*, thapsigargin (TG), a well-known activator of SOC induced robust inward currents, which were greatly reversed by La^3+^ (2 μM), an inhibitor of SOC (25, 30). Angiotensin II (Ang II) plays an important role in podocyte physiology and pathology (9, 10). Ang II-stimulated Ca^2+^ signaling involves several type of Ca^2+^ channels, including SOC and receptor-operated Ca^2+^ channels or TRPC channels (9, 22, 25). To determine the physiological and pathological relevance of SOC in podocytes, we also measured SOC currents in response to Ang II. As shown in Figure 1, *D* and *E*, Ang II treatment (1 μM) evoked robust inward currents. The Ang II-induced currents were significantly, but not completely, blocked by 3,5-bis(trifluoromethyl) pyrazole (BTP) 2 (10 μM), a SOC blocker, suggesting a component of SOC currents. The residue currents in the presence of BTP2 might be attributed to TRPC channels.

Taken together, these data suggest that podocytes express functional SOCs.

### HG treatment increased Orai1 but not STIM1 protein abundance in HPCs

**Figure 2:**
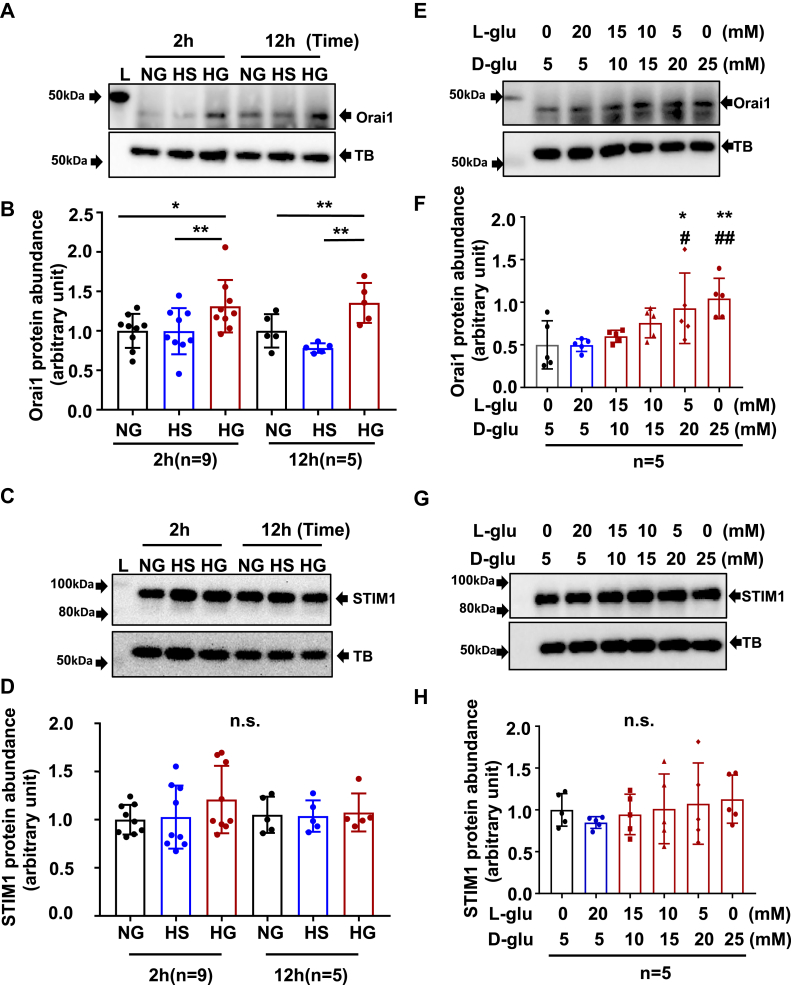
**HG treatment increased Orai1, but not STIM1 protein abundance in HPCs.***A* and *B*, representative Western blots, showing Orai1 protein abundance in HPCs treated with HG for different time periods and treated with different glucose concentrations for 12 h. *A*, fully differentiated podocytes were cultured in 0.5% FBS medium containing NG (5.6 mM D-glucose), HS (5.6 D-glucose + 20 mM L-glucose), or HG (25 mM D-glucose) for 2 and 12 h. α-Tubulin (TB) was used as a loading control. *B*, fully differentiated podocytes were cultured in 0.5% FBS medium containing 5, 10, 15, 20, and 25 mM D-glucose (D-glu) for 12 h. Appropriate concentration of L-glucose (L-glu) was added to adjust osmolality of all culture medium to 25 mmol/l. *C* and *D*, summary data from experiments presented in (*A*) and (*B*), respectively. Protein abundance level in each group was normalized to TB and then the values in each group were further normalized to those of the NG group. “n” indicates the number of independent experiments, ∗*p* < 0.05, ∗∗*p* < 0.01, ns: no significant difference. One-way ANOVA followed by Tukey’s multiple comparison test. *E* and F, representative Western blots, showing STIM1 protein abundance in HPCs treated with HG (25 mM) for different time periods and treated with different glucose concentrations for 12 h. The treatment protocols were the same as Figure 2, *A* and *B*. *G* and *H*, summary data from experiments presented in (*E*) and (*F*), respectively. “n” indicates the number of independent experiments, ∗*p* < 0.05, ∗∗*p* < 0.01, compared with NG; #*p* < 0.05*, ##p* < 0.01, compared with HS. ns: no significant difference, one-way ANOVA followed by Tukey’s multiple comparison test. FBS, fetal bovine serum; HG, high glucose; HPC, human podocyte; HS, high osmolality; NG, normal glucose; STIM1, stromal interaction molecule 1.

To explore associations of SOCE signaling in podocytes with DN, we conducted Western blot and evaluated Orai1 protein abundance in response to HG, a known pathogenic factor to trigger podocyte injury and development of DN (31) in HPCs. As shown in Figure 2, *A*–*D*, HG treatment significantly increased abundance of Orai1 protein. The HG response occurred as early as 2 h treatment and sustained for at least 12 h (Fig. 2, *A* and *C*). Furthermore, HG increased Orai1 protein content in a dose-dependent manner (Fig. 2, *B* and *D*). The Orai1 response in HPCs was specific to HG because this response was not observed in the cells with osmotic CT.

STIM1 protein is another essential component of SOCE signaling. We also performed Western blot in cultured HPCs and assessed HG effect on STIM1 protein abundance. Unlike Orai1, STIM1 protein levels were not significantly changed by HG treatment at the same time periods (Fig. 2, *E* and *G*) and glucose concentrations (Fig. 2, *F* and *H*) as those for Orai1.

### HG treatment enhanced SOCE in HPCs

**Figure 3:**
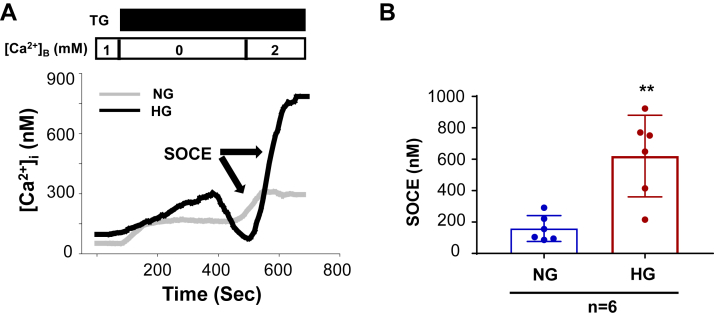
**HG treatment enhanced SOCE in HPCs**. *A*, representative traces, showing TG (1 μM)-evoked Ca^2+^ response in HPC with HG and NG treatment for 12 h. [Ca^2+^]B represents the Ca^2+^ concentration in bathing solution. [Ca2+]i represents the intracellular Ca^2+^ concentration. SOCE was the increase in [Ca^2+^]i upon switching the bathing solution from Ca2+ free to 2 mM Ca^2+^ solution. The numbers inside the *horizontal bar* on top of each graph indicate Ca^2+^ concentration in bathing solution. *B*, statistical analysis of SOCE in HPC with HG and NG treatment. “n” indicates the number of cells analyzed in each group, ∗∗*p*< 0.01, compared to NG group, unpaired *t* test. HG, high glucose; HPC, human podocyte; NG, normal glucose; SOCE, store-operated Ca2+ entry.

To evaluate the functional change of the podocyte Orai1 channels in response to HG treatment, we measured the Fura-2 fluorescence-indicated SOCE response using the classic “Ca^2+^ add-back” protocol described in our previous publications in podocytes with and without HG treatment (25, 32). As shown in Figure 3, TG (1 μM) evoked a transient of [Ca^2+^]_i_ in 0 mM Ca^2+^ bathing solution in HPCs both with and without HG treatment. This initial response indicates the ER Ca^2+^ store depletion by TG, which is the trigger of SOC opening. When replacing the Ca^2+^-free bathing solution with 2 mM Ca^2+^ solution, [Ca^2+^]_i_ rose. The amplitude of the [Ca^2+^]_i_ elevation indicated SOCE (30). In agreement with Western blot results (Fig. 2, *A*–*D*), SOCE was significantly increased in HPCs with HG treatment (25 mM for 12 h) compared with HPCs without HG treatment. Thus, the calcium imaging data provided functional evidence supporting that SOCE was enhanced in podocytes exposed to ambient HG.

### SOCE inhibition blunted HG-induced reduction of nephrin protein abundance in HPCs

**Figure 4:**
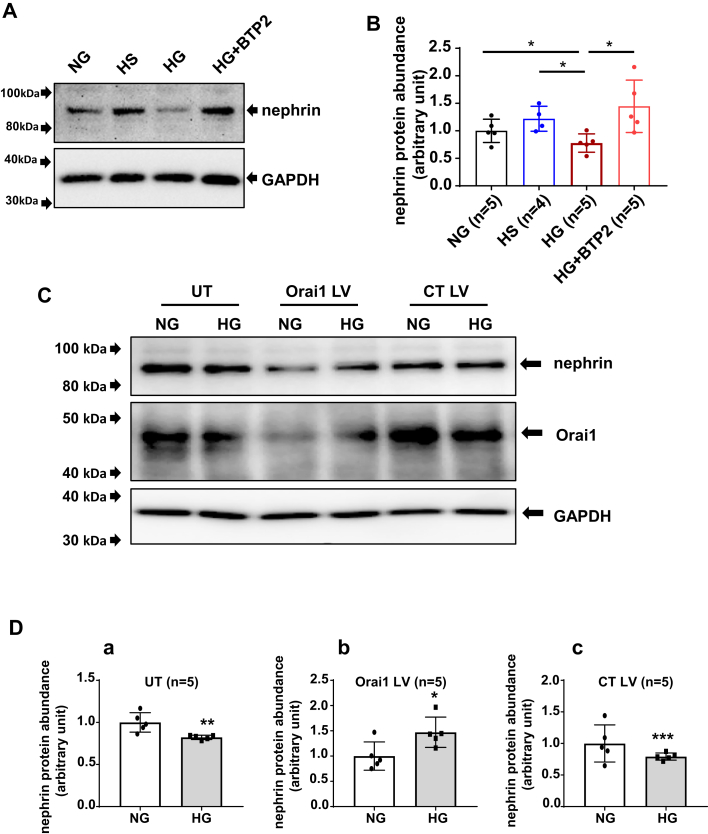
**SOCE inhibition blunted HG-induced nephrin protein reduction in HPCs**. *A*, representative Western blot showing nephrin protein abundance in HPCs with and without HG treatment in the absence and presence of SOC inhibitor BTP2. Fully differentiated podocytes were cultured in 0.5% FBS medium containing NG, HS, HG, and HG + BTP2 (4 μM) for 24 h. GAPDH was used as loading control. *B*, summary data from experiments presented in (*A*). “n” indicates the number of independent experiments, ∗*p*< 0.05, one-way ANOVA followed by Tukey’s multiple comparison test. *C*, representative Western blot showing nephrin and Orai1 protein abundances in response to HG treatment in HPCs without virus infection (UT) or with infection with Orai1 CRISPR-Cas9 lentivirus (Orai1 LV) or control lentivirus (CT LV). GAPDH was used as the loading control. The podocytes in Orai1 LV and CT LV groups were selected by puromycin. The three groups of cells were further divided into two subgroups. One subgroup was incubated with NG medium and the other subgroup was treated with HG without or after puromycin selection. *D*, summary data showing the difference in nephrin protein abundance in podocytes without (a) and with treatment of CRISPR-Orai1 virus (b) and control virus (c). “n” indicates the number of independent experiments. ∗*p*< 0.05, ∗∗*p*< 0.01, ∗∗∗*p* < 0.001, NG *versus* HG (Paired *t* test). BTP, bis(trifluoromethyl) pyrazole; Cas9, CRISPR-associated protein 9; HG, high glucose; HPC, human podocyte; NG, normal glucose; SOCE, store-operated Ca2+ entry.

Indications of podocyte injury include loss of podocyte phenotype protein markers, such as nephrin, podocin, and synaptopodin (33, 34). We therefore examined if HG treatment decreased abundance of those marker proteins and if SOCE was involved in the responses. As shown in Figure 4, HG treatment (25 mM for 24 h) but not the osmotic CT (high osmolarity [HS]) significantly decreased nephrin protein content in HPCs. However, this HG effect was not observed in the presence of BTP2 (4 μM), a selective SOC blocker (Fig. 4, *A* and *B*). Similarly, deletion of Orai1 using CRISPR-Cas9 approach also abolished the HG-induced decrease in nephrin protein abundance (Fig. 4, *C* and *D*).

We also evaluated HG effects on podocin and synaptopodin, the other two podocyte phenotype marker proteins. However, no significant changes in abundance of the two proteins were observed in HPCs with and without HG treatment (25 mM for 24 h) (Fig. S1).

### SOCE inhibition blunted HG-induced HPCs cytoskeleton dysregulation

**Figure 5:**
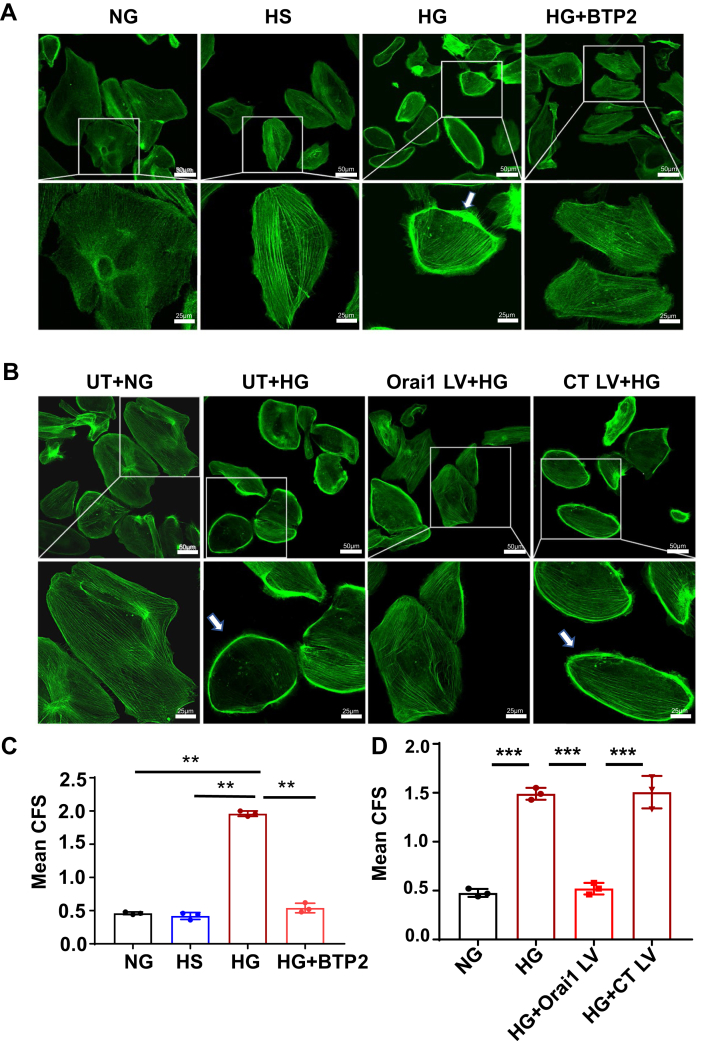
**SOCE inhibition blunted HG-induced podocyte cytoskeleton disorganization**. *A*, representative immunofluorescence staining of F-actin. Fully differentiated HPCs were cultured in 0.5% FBS medium containing NG, HS, HG, and HG + BTP2 (4 μM) for 7 days. In NG and HS group, F-actins were distributed as homogenous bundles that traversed the cells along the axis of the podocytes. The *white arrow* shows the ring of cortical F-actin formation and central stress fiber loss, which was recognized as F-actin rearrangement. Scale bar in upper panels represent 50 μm and at bottom panels represent 25 μm. *B*, representative immunofluorescence staining of F-actin in HPCs incubated with NG without virus treatment (UT + NG), with HG without virus treatment (UT + HG), infected with Orai1 CRISPR-Cas9 lentivirus followed by HG treatment (Orai1 LV + HG), and infected with EGFP control lentivirus followed by HG treatment (CT LV + HG). Scale bar in upper panels represent 50 μm and at bottom panels represent 25 μm. The *white arrow* shows the ring of cortical F-actin formation and central stress fiber loss. *C*, statistical analysis of cytoskeleton reorganization. The mean cortical F-actin score (CFS) = total scores/cells in each group. Each *dot* indicates the mean CFS of podocytes in one slide. A minimum of 50 cells was counted in randomly selected six images in one slide. The experiment was repeated three times. ∗∗*p* < 0.01, one-way ANOVA followed by Tukey’s multiple comparison test. *D*, statistical analysis of cytoskeleton reorganization from experiments presented in (*B*). A minimum of 30 cells were analyzed in randomly selected six images in one slide. The experiment was repeated three times. ∗∗∗*p* < 0.001, one-way ANOVA followed by Tukey’s multiple comparison test. Cas9, CRISPR-associated protein 9; FBS, fetal bovine serum; HG, high glucose; HPC, human podocyte; LV, lentivirus; NG, normal glucose; SOCE, store-operated Ca2+ entry.

Podocyte function and foot process mobility are highly dependent on the actin cytoskeleton. No matter what the initial cause is, the final pathway for podocyte injury is cytoskeleton dysfunction and rearrangement (1). Studies have shown that HG disrupts podocyte cytoskeleton, which is characterized by central stress fiber disorganization and increased assembly of F-actin in cortical regions (34, 35). To determine whether SOCE contributed to the HG-induced impairment of podocyte cytoskeleton, we assessed podocyte F-actin arrangement in response to HG treatment with and without blockade of SOCE. Alexa Fluor 488 Phalloidin was used to stain F-actin. As shown in Figure 5, F-actins in the podocytes cultured in normal glucose (NG) medium were distributed as homogenous bundles that traversed the cells along the axis of podocytes. HG, but not the osmotic CT (HS) treatment, resulted in loss of central stress fibers and significantly increased formation of cortical F-actin. The cytoskeleton disorganization and increase in cortical F-actin were significantly blunted by BTP2 (4 μM). To further determine the role of Orai1 channels in HG-induced podocyte cytoskeleton disorganization, we constructed *orai1* KO HPCs using CRISPR-Cas9 LV (Fig. 5*C*) and evaluated F-actin organization in response to HG treatment. In agreement with the results presented previously, HG treatment failed to disrupt cytoskeleton and form the cortical F-actins in HPCs infected with Orai1 CRISPR-Cas9 LV, but did in HPCs with CT LV infection (Fig. 5, *B* and *D*). These results suggest that SOCE signaling mediated HG-induced podocyte cytoskeleton disruption.

### SOCE mediated HG-induced calpain activation in HPCs

**Figure 6:**
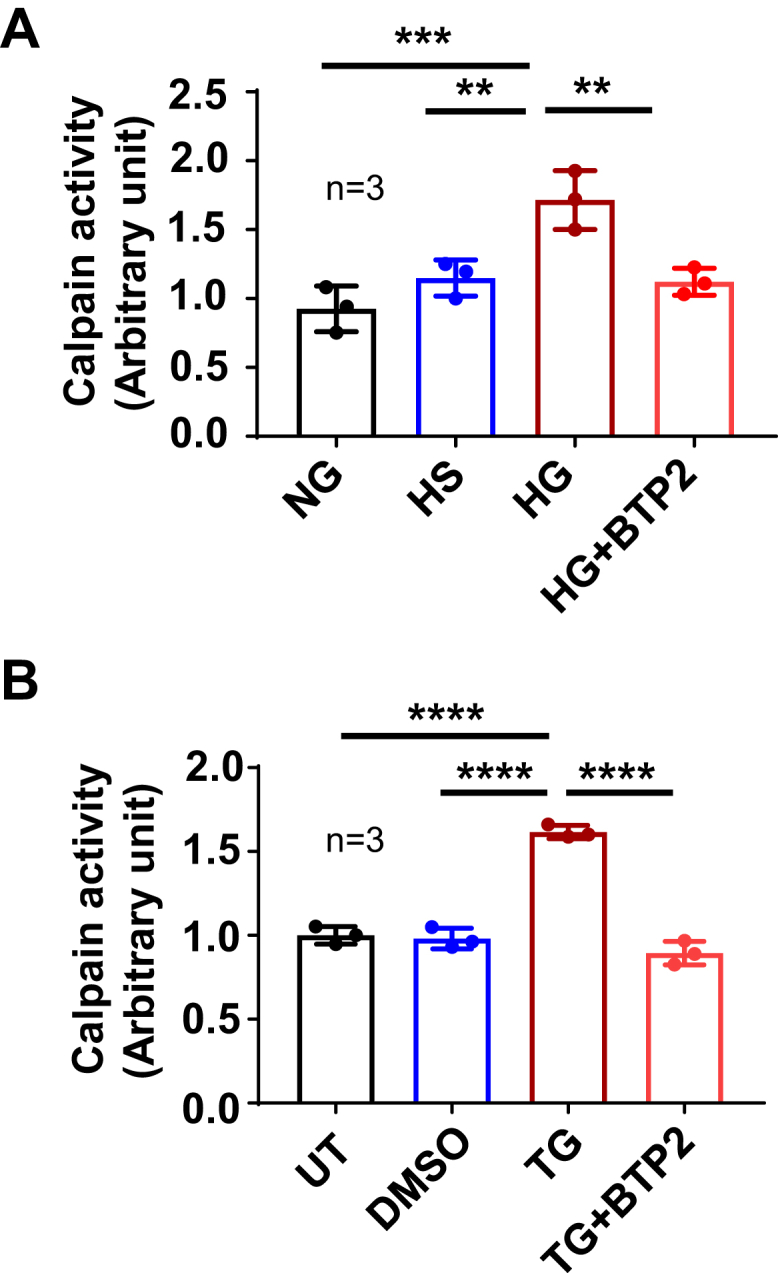
**SOCE mediated HG-induced activation of calpain in HPCs**. *A*, calpain activity in HPCs incubated with NG or with osmotic control (HS) or with HG or with HG plus 4 μM BTP2 (HG + BTP2). Calpain activity in each measurement was normalized to the total amount of protein. This value in each group was further normalized to that in NG group. “n” indicates the number of independent experiments. ∗∗∗*p* < 0.001, ∗∗*p* < 0.01, ∗∗*p* < 0.01, one-way ANOVA repeated measurement followed by Tukey’s multiple comparison test. *B*, the calpain activity in podocytes without any treatment (UT) or treated with DMSO (vehicle control, 1:1000), or TG (1 μM), or TG plus 10 μM BTP2 (TG + BTP2). Calpain activity in each measurement was normalized to the total amount of protein. This value in each group was further normalized to that in UT group. “n” indicates the number of independent experiments, ∗∗∗∗*p* < 0.0001, one-way ANOVA repeated measurement followed by Tukey’s multiple comparison test. BTP, bis(trifluoromethyl) pyrazole; HG, high glucose; HPC, human podocyte; HS, high osmolality; LV, lentivirus; NG, normal glucose; SOCE, store-operated Ca2+ entry.

Calpains are the family of Ca^2+^-activated cysteine proteases, which have high sensitivity to Ca^2+^ and are mainly activated by increased intracellular Ca^2+^ (23). Recent studies also showed that calpain activity regulates podocyte cytoskeleton organization and motility (36, 37). Therefore, we reasoned that activation of calpain signaling was a downstream mechanism for SOCE-induced podocyte injury in the HG condition. To test this speculation, we first examined the podocyte calpain activity with or without HG treatment. As shown in Figure 6*A*, HG (25 mM D-glucose) but not its osmotic CT (5.6 mM D-glucose + 20 mM L-glucose) significantly increased podocyte calpain activity. To determine if this increased calpain activity by HG treatment was mediated by SOCE activation, we repeated the experiment under condition of SOCE inhibition. In the presence of BTP2, HG treatment failed to increase calpain activity anymore (Fig. 6*A*).

We next examined the direct effect of SOCE on calpain activity. As shown in Figure 6*B*, TG (1 μM) but not its vehicle CT (dimethyl sulfoxide at 1:1000) significantly increased calpain activity in HPCs. The TG response was significantly blunted by BTP2 (10 μM), suggesting the SOCE mechanism in the TG-activated calpain. Taken together, these results suggest that HG activated calpain signaling through activation of SOCE in HPCs.

### Calpain activity inhibition blunted HG-induced nephrin protein reduction in HPCs

**Figure 7:**
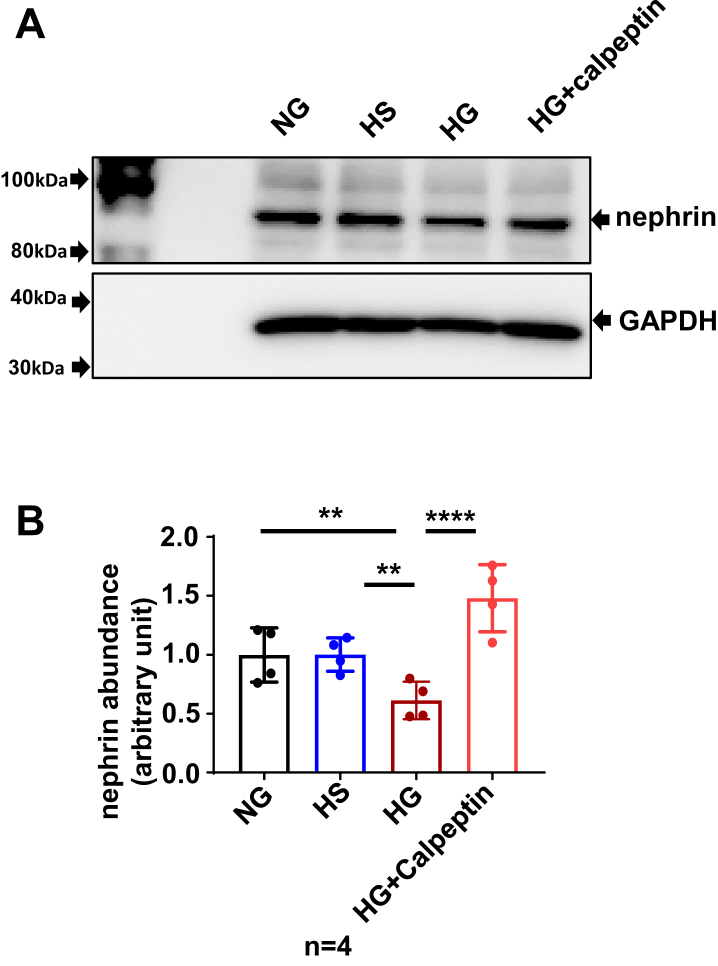
**Calpain inhibition blocked HG-induced nephrin protein reduction in HPCs**. *A*, representative Western blot. Fully differentiated podocytes were incubated with NG, or osmotic control (HS), or HG or HG with 5 μM calpeptin (HG + calpeptin) for 24 h. GAPDH was used as a loading control. *B*, summary data. ∗∗*p* < 0.01, ∗∗∗∗*p* < 0.0001, “n” indicates the number of independent experiments, one-way ANOVA repeated measurement followed by Tukey’s multiple comparison test. HG, high glucose; HPC, human podocyte; HS, high osmolality; NG, normal glucose.

To further determine if activation of SOCE/calpain pathway mediated HG-induced podocyte injury, we used calpeptin (5 μM), a calpain inhibitor to inhibit calpain activity in the presence of HG and evaluated the resultant effect on the nephrin protein abundance. Consistent with data presented in Figure 4, HG treatment for 24 h, but not its osmotic CT, significantly decreased nephrin protein abundance. However, calpeptin completely blocked the HG-induced response (Fig. 7).

## Discussion

In this study, we evaluated the detrimental mechanisms of overwhelming Orai1/SOCE/calpain signaling in ambient HG-induced podocyte injury. Our results suggest that inhibition of SOCE/calpain signaling pathway can protect podocytes against injury induced by HG, a major contributor to the onset and development of DN.

The structural and functional integrity of podocytes are dependent on intracellular Ca^2+^ homeostasis. In this regard, the importance of TRPC6 and TRPC5 Ca^2+^ channels in maintaining podocyte physiology has been well studied. Gain of function of TRPC6 mutations in podocytes resulted in renal diseases (7, 38). TRPC6 activation in podocytes causes required proteinuria kidney diseases and mediates Ang II-induced podocyte injury (8, 9, 10). Dysfunction of TRPC5 channel also caused podocyte damage (6, 39). In the present study, we reported another Ca^2+^ channel signaling, SOCE, as another mechanism contributing to podocyte injury related to diabetes mellitus. We showed biochemical and functional evidence that this Ca^2+^ signaling in podocytes was upregulated by HG. Most importantly, pharmacological or/and biological inhibition on SOCE significantly blunted HG-induced podocyte injury. Apparently, podocyte Ca^2+^ homeostasis is controlled by multiple Ca^2+^ channel mechanisms and SOCE is a component of the family. A disturbance of the homeostasis derived from supernormal SOCE plays an indispensable role in podocyte injury and renal injury. In support, one recent study by Mehrotra *et al.* reported that oral administration of BTP2 improved renal function and ameliorated renal fibrosis in high-salt diet-induced chronic kidney disease rats (40).

It is known that both Orai1 and STIM1 are the essential components of SOCE pathway (12, 13, 14, 15, 16, 17, 18, 19). Activation of SOCE requires the physical interactions between the two proteins at STIM1–Orai1 stoichiometry ranging from 1:1 to 2:1 (41). In the present study, we found that HG enhanced SOCE by increasing abundance of Orai1 without change in amount of STIM1 protein. It may be argued that with the interaction ratio of 1:1 or 2:1 of STIM1:Orai1, no increase in number of STIM1 molecules should not activate more Orai1 channels and thus should not increase SOCE. However, the interaction of STIM1 with Orai1 is not only determined by amount of the protein but also the activity of STIM1. For instance, STIM1 interaction with Orai1 is tightly modulated by specific residue phosphorylation (such as Ser^575^, Ser^608^, and Ser^621^) (42). One recent study found that glucose-induced fluctuation in O-GlcNAcylation can modulate STIM1 activation in HEK cells. *O*-GlcNAcylation of STIM1 at Thr^626^ increased its interaction with Orai1, probably *via* regulation of Ser^621^ phosphorylation (43). Therefore, it is possible that a portion of STIM1 proteins in podocytes are not active under normal condition (*e.g.*, NG environment) but are activated through posttranslational modifications under the environment of HG. Another simple explanation is that podocytes contain more STIM1 molecules than Orai1 molecules for the 1:1 or 2:1 interactions, and HG treatment stimulates the spare STIM1 proteins interacting with additional Orai1 proteins induced by HG treatment.

A recent study by Jin *et al.* reported that the STIM1 protein abundance was significantly increased in mouse podocytes cultured with serum from rats with DN (44). Their findings do not contradict with our findings in the present study. The increase in STIM1 protein content in their study might not be attributed to hyperglycemia but to the hormones, growth factors, and/or transcription factors, the serum levels of which are elevated in diabetes.

We noticed that in the present study, HG induced molecular/event changes in podocytes sequentially in terms of time periods. For instance, an increase in Orai1 protein abundance and SOCE required at least 2 h treatment, but a decrease in nephrin protein content needed a minimum of 24 h exposure to HG, and the cortical F-actin formation even took about 7 days with HG treatment. This temporal sequence of the biochemical/functional/structural changes suggest a serial chain of signaling pathway initiated by HG, that is, HG/Orai1/SOCE/nephrin/cytoskeleton. It is understandable that each step in this chain requires certain period of time dependent on the nature of the biological processes.

In addition to nephrin, changes in several other podocyte phenotype marker proteins, such as podocin and synaptopodin, are also associated with podocyte injury (45). However, we did not find a significant decrease in the content of both podocin and synaptopodin proteins under the experimental conditions in the present study (25 mM glucose for 24 h). One possibility is that nephrin is more susceptible to a change in glucose concentration and the other two proteins are more tolerant to HG treatment. Supportedly, one recent study demonstrated that HG (22 mM) treatment for 7 days significantly decreased podocin protein abundance in HPCs (46). Thus, nephrin might be an early indicator of podocyte injury. Indeed, a recent study reported that the urinary level of nephrin-positive podocyte-derived extracellular vesicles was a biomarker of early podocyte injury (47).

CRISPR-Cas9 technique was used to delete Orai1 in podocytes in this study. Since podocytes are terminally differentiated cells and are much easier to be infected by LV (48), we used LV as the vehicle to deliver the CRISPR-Cas9 system. Our study showed that with CRISPR-Cas9–Orai1 LV treatment, HG failed to decrease nephrin protein abundance. In fact, the level of the protein was significantly increased (Fig. 4, *C* and *D*). These results suggest that SOCE not only mediates HG-induced loss of nephrin protein but also has a tonic inhibition on the protein dynamics.

We showed that calpain was the effector of SOCE in podocytes, mediating SOCE-induced nephrin protein reduction. Calpains are intracellular Ca^2+^-activated cysteine proteases, which are mainly activated by increased intracellular Ca^2+^ (23). Activation of calpain signaling was recently demonstrated to mediate Ang II-induced podocyte injury (49). Podocytes possess multiple Ca^2+^ entry pathways and different Ca^2+^ signals may have distinct downstream effectors. For instance, TRPC6-mediated Ca^2+^ influx upregulates the calcium-dependent calcineurin/nuclear factor of activated T cells pathway (50, 51). TRPC5-mediated Ca^2+^ influx acts on synaptopodin and Rac1 (5, 6). However, the Ca^2+^-dependent proteinase calpain seems to be a master effector serving multiple Ca^2+^ signaling pathways. In addition to SOCE pathway reported by the present study, both TRPC5 and TRPC6 Ca^2+^ signals also activate calpain pathway (36, 37, 52). Thus, Ca^2+^ signals from different sources in podocytes may interact with each other at a site of a particular signaling pathway. Another example is the finding from a recent study that Orai1-mediated SOCE induces podocyte actin remodeling by activation of calcineurin pathway (11), a pathway also activated by TRPC6-mediated Ca^2+^ signals (50, 51). One advantage of sharing the same effector among different Ca^2+^ pathways is that the Ca^2+^ signals from different sources converge on one molecule where all signals are integrated to a final message delivered to next event. Because podocyte injury in many kidney diseases results from overloading intracellular Ca^2+^ due to abnormal activation of various Ca^2+^ channels and inhibition on calpain, the common effector of different Ca^2+^ signaling pathways may be a therapeutic option, as suggested by a recent study (49).

The present study shows that HG-induced SOCE activation contributes to podocyte calpain activation. Among 15 subtypes, calpain 1, 2, 4, 6, 10, and 15 are expressed in podocytes (37). The present study showed that the HG-induced podocyte injury was ameliorated by calpeptin, which is a calpain-1/calpain-2 inhibitor. Thereby, the subtypes of calpain-1 and calpain-2 might be the major calpains involved in HG/SOCE/podocyte injury cascade. Our results are consistent with a recent study, which revealed that inhibition of calpain-1/calpain-2 or KO of *capn1/2* reduced proteinuria and stabilized podocyte adhesion in mice with chronic kidney disease (53).

It should be noted that store-operated Ca^2+^ signaling–induced cell responses can be mediated by multiple signaling pathways, such as calcineurin (11) and TGF-β1/Smad (20, 54, 55) pathways. Our findings in this study do not exclude a possible involvement of a calpain-independent mechanism in SOCE-induced podocyte injury.

## Conclusion

Collectively, the present study identified a previously unrecognized cascade of HG/Orai1/SOCE/calpain in podocytes, upregulation of which induces podocyte injury manifested by loss of nephrin and disorganization of cytoskeleton. Podocyte injury is an early feature of DN and exacerbates the devastated kidney disease. Our findings suggest that inhibition of SOCE and/or calpain signaling pathway may be a therapeutic option for DN.

## Experimental procedures

### Key resources table

**Table:** 

Reagent	Source	Identifier
Antibodies for Western blots		
Rabbit polyclonal anti-Orai1	Sigma–Aldrich	Cat#: O8264
Rabbit polyclonal antipodocin	Sigma–Aldrich	Cat#: P0372
Rabbit polyclonal antinephrin	Sigma–Aldrich	Cat#: PRS2265
Rabbit polyclonal anti-STIM1	Proteintech	Cat#: 11565-1-AP
Rabbit polyclonal antisynaptopodin	NOVUS	Cat#: NBP2-39100
Mouse monoclonal anti-GAPDH	EMD Millipore	Cat#: MAB374
Mouse monoclonal anti-α-tubulin	Santa Cruz Biotechnology	Cat#: sc-5286
goat anti-rabbit Ig HRP	Invitrogen	Cat#: 31460
goat antimouse Ig HRP	Invitrogen	Cat#: 31430
Reagents for calcium imaging		
Thapsigargin	Sigma–Aldrich	Cat#: 586005
Fura 2-AM	Invitrogen	Cat# F1221
Pluronic F-127	Invitrogen	P6867
EGTA	Sigma–Aldrich	E-0396
Ionomycin	Sigma–Aldrich	Cat# I3909
Reagents for F-actin staining		
Alexa Fluor 488 Phalloidin	Thermo Fisher Scientific	Cat# A12379
TritonX-100	Sigma–Aldrich	Cat#: T9284
Mounting medium with DAPI	VECTOR	H1200
Commercial kits and reagents for Calpain activity assay		
Calpain activity assay kit	Abcam	Cat#: ab65308
Coomassie-based protein assay	Thermo Fisher Scientific	Cat#: PI23236
Cell culture reagents		
RPMI1640 medium	Gibco	Cat#: 11875093
Fetal bovine serum (FBS)	Gibco	Cat#: 10082147
Insulin, transferrin, and selenium (ITS)	Sigma–Aldrich	Cat#: I-3146
RPMI1640 medium glucose free	Sigma–Aldrich	Cat#: R1383
D-glucose	Sigma–Aldrich	CAS#: 50-99-7
L-glucose	Sigma–Aldrich	G5500
Penicillin-Streptomycin (10,000 U/ml)	Gibco	Cat#: 15140122
Other reagents, chemicals, and experiment materials		
BTP2 (YM58483)	Tocris	Cat#: 3939
Protease/phosphatase inhibitor	Sigma–Aldrich	Cat#: 11697498001
Polyvinylidene fluoride (PVDF) membranes	Millipore	IPVH00010
Calpeptin	Sigma–Aldrich	Cat#: C8999
Pico luminol/enhancer solution	Thermo Scientific	Cat#: 34580
SuperSignal West Femto	Thermo Scientific	Cat#: 34095

Abbreviations: Cat, catalog; DAPI, 4',6-diamidino-2-phenylindole; HRP, horse radish peroxidase; Ig, immunoglobulin.

#### Cell culture

Immortalized HPCs were obtained from Dr Jochen Reiser (Rush University) and cultured following the protocols they provided (56). HPCs were cultured in 75 cm^2^ flask with RPMI1640 medium (Catalog no.: 11875093; Gibco) containing 10% fetal bovine serum (FBS) (Gibco), 100 U/ml penicillin, 100 μg/ml streptomycin, 1 × insulin, transferrin, and selenium (ITS) (Catalog no.: I-3146, Sigma–Aldrich) in a 33 °C with 5% CO_2_ incubator for proliferation. When HPCs reached approximately 60% confluence, they were moved to 37 °C for 10 to 14 days for differentiation. Cell culture media were changed three times per week. Fully differentiated podocytes were growth arrested with serum free (0.5% FBS) media overnight prior to various experimental treatments as specified in figure legends. Based on experimental design, the cell culture media had three different concentrations of glucose as follows unless specified in figure legend: (1) 5.6 mM D-glucose, designated as NG; (2) 25 mM D-glucose, designated as HG; and (3) 5.6 mM D-glucose + 20 mM L-glucose, designated as HS CT.

#### CRISPR-Cas9 LV infection

The Orai1 CRISPR LV (pLV[CRISPR]-hCas9:T2A:Puro-U6>hORAI1[gRNA#117]) and EGFP CT LV (pLV[Exp]-EGFP:T2A:Puro- F1A>mCherry) were designed by ourselves using the VectorBuilder online platform and were generated by VectorBuilder Inc. The viruses were puromycin resistant. Fully differentiated HPCs in a total number of about 13 × 10^4^ were infected with orai1 CRISPR-Cas9 LV or CT LV at 40 multiplicity of infection in RPMI media with 2% FBS without penicillin/streptomycin for 48 h. Polybrene (5 μg/ml) was used to increase the infection efficiency. Then, the culture media were changed to complete RPMI media with 10% FBS. Two days later, puromycin at the concentration of 2 μg/ml was added to the culture medium for selecting positively infected HPCs. The Orai1-deleted HPCs were randomly undertaken with different treatments as indicated in figure legends.

#### Western blot analysis

Western blot was performed as described in our previous publication (57). Proteins were extracted from cells using the lysis buffer containing protease/phosphatase inhibitor (Sigma–Aldrich). Equal amount of proteins (30 μg per lane) were separated by 10% SDS-PAGE, transferred to polyvinylidene fluoride membranes (Millipore), and probed with primary antibodies at 4 °C overnight (anti-Orai1, 1:800; anti-STIM1, 1:2000; anti-nephrin, 1:1000; anti-synaptopodin, 1:800; anti-podocin, 1:1000; anti-GAPDH, 1:300; and anti-α-tubulin, 1:200). After washing membranes with Tris-buffered saline with Tween for three times, the membranes were then incubated with secondary antibodies (1:5000) in room temperature (RT) for 1 h. Orai1 protein band was developed with Super Signal West Femto, but all other protein bands were developed with Pico Luminol/Enhancer Solution (Thermo Scientific). The bands were visualized and captured using the AlphaEase FC Imaging system (Alpha Innotech). The band density was measured by AlphaEase FC software (Alpha Innotech) as previously described (57).

#### Electrophysiology

The conventional whole-cell voltage-clamp configuration was performed in single podocyte at RTwith a Warner PC-505B amplifier (Warner Instruments) and Clampex 9.2 (Axon Instruments). Glass pipettes (plain, Fisher Scientific) with resistances of 3 to 5 MΩ were prepared with a pipette puller and polisher (PP-830 and MF-830, respectively, Narishige). When the whole-cell configuration was achieved, cell capacitance and series resistance were immediately compensated. The whole-cell currents were continuously recorded at a holding potential of −80 mV until the end of each experiment (10–15 min). Current traces were filtered at 1 kHz and analyzed offline with Clampfit 9.4 (Axon Instruments). The compositions of the pipette solution were (in mM): 135 Cs-aspartate, 6 MgCl_2_, and 10 Hepes, pH 7.2. The compositions of the bathing solution were (in mM): 120 NaCl, 5 KCl, 1 MgCl_2_, 10 CaCl_2_, 10 Hepes, pH 7.4.

#### Calcium imaging

Fura 2 fluorescence-indicated Ca^2+^ entry was measured to evaluate SOCE in HPCs as described previously (25). Briefly, HPCs, plated on a coverslip were loaded with 5 μM fura 2-AM plus 0.018 g/dl pluronic F-127 for 45 min followed by a 20 min incubation in fura-2 free physiological saline solution in dark at RT. The coverslip was then mounted to a perfusion chamber and placed on the stage of a Nikon Diaphot inverted microscope. NIS Elements AR software (Nikon Instrument) was used to monitor the 340 and 380 nm excitation wavelength as well as 510 nm emission wavelength in dark room at RT. [Ca^2+^]_i_ was calculated using the software following the manufacturer’s instruction. Calibrations were performed at the end of each experiment by inducing the maximum calcium entrance using 5 μM ionomycin, followed by a calcium chelator EGTA (5 mM).

#### Staining of the actin cytoskeleton

HPCs were washed with prewarmed PBS two times before fixed with 4% paraformaldehyde at RT for 15 min. Then, the HPCs were rinsed three times with PBS, followed by permeabilization in 0.1% Triton X-100 in PBS for 15 min. Alexa Fluor 488 Phalloidin for F-actin staining was dissolved in 150 μl dimethyl sulfoxide to yield a 400× stock solution at a concentration of 2000 assays/ml, which is equivalent to approximately 66 μM. The cells were then stained with Phalloidin and visualized using a confocal microscope (Olympus). To evaluate normality of cytoskeleton arrangement, the cortical F-actin in each podocyte was scored by an observer blind to the experimental treatments. The scores of the cortical F-actin in cells were assigned into 1 to 4 according to the degree of cortical F-actin formation and central stress fiber absence as follows: 0, no cortical F-actin, normal stress fibers; 1, cortical F-actin deposits on less than 1/2 of the cell border; 2, cortical F-actin deposits on more than 1/2 of the cell border; and 3, complete cortical ring formatting or total absence of central stress fiber.

#### Calpain activity assays

Calpain activity was determined according to the manufacturer’s protocol (Abcam). HPCs (approximately 2 × 10^6^) were seeded into a 10 mm dish. After treatment, cells were harvested and resuspended in extraction buffer and incubated on ice for 20 min. Samples were then centrifuged for 1 min in a microcentrifuge (10,000*g*) at 4 °C, and the supernatant was transferred into a clean tube. A volume of 85 μl supernatant was then transferred to a 96-well plate and incubated with 5 μl calpain substrates together with 10 μl reaction buffer for 1 h at 37 °C protected from light before reading the fluorescence absorbance at Ex/Em = 400/505 nm. The absorbances were then normalized to sample protein concentration measured by Coomassie-based protein assay (Cat# PI23236; Thermo Fisher Scientific).

### Statistical analysis

All quantitative data passed Shapiro–Wilk normality test before performing parametric test. All values were presented as means ± SD and were analyzed using GraphPad Prism 7. For comparisons between two independent groups, unpaired Student’s *t* tests were performed; for comparisons of multiple groups, one-way ANOVA followed by Tukey’s multiple comparison test were performed. Comparisons between treatment within one group was analyzed by paired *t* test. Results with *p* value of less than 0.05 were considered statistically significant.

## Data availability

All data are included in the article.

## Supporting information

The article contains supporting information.

## Conflict of interests

The authors declare that they have no conflicts of interest with the contents of this article.

## Supporting information

Figure-S1-revision
