# Supplementary figures and images for: Enhanced Orai1-mediated store-operated Ca2+ channel/calpain signaling contributes to high glucose-induced podocyte injury
Source: J Biol Chem. 2022 Apr 29;298(6):101990. doi: 10.1016/j.jbc.2022.101990 (PMC9136128; doi:10.1016/j.jbc.2022.101990)

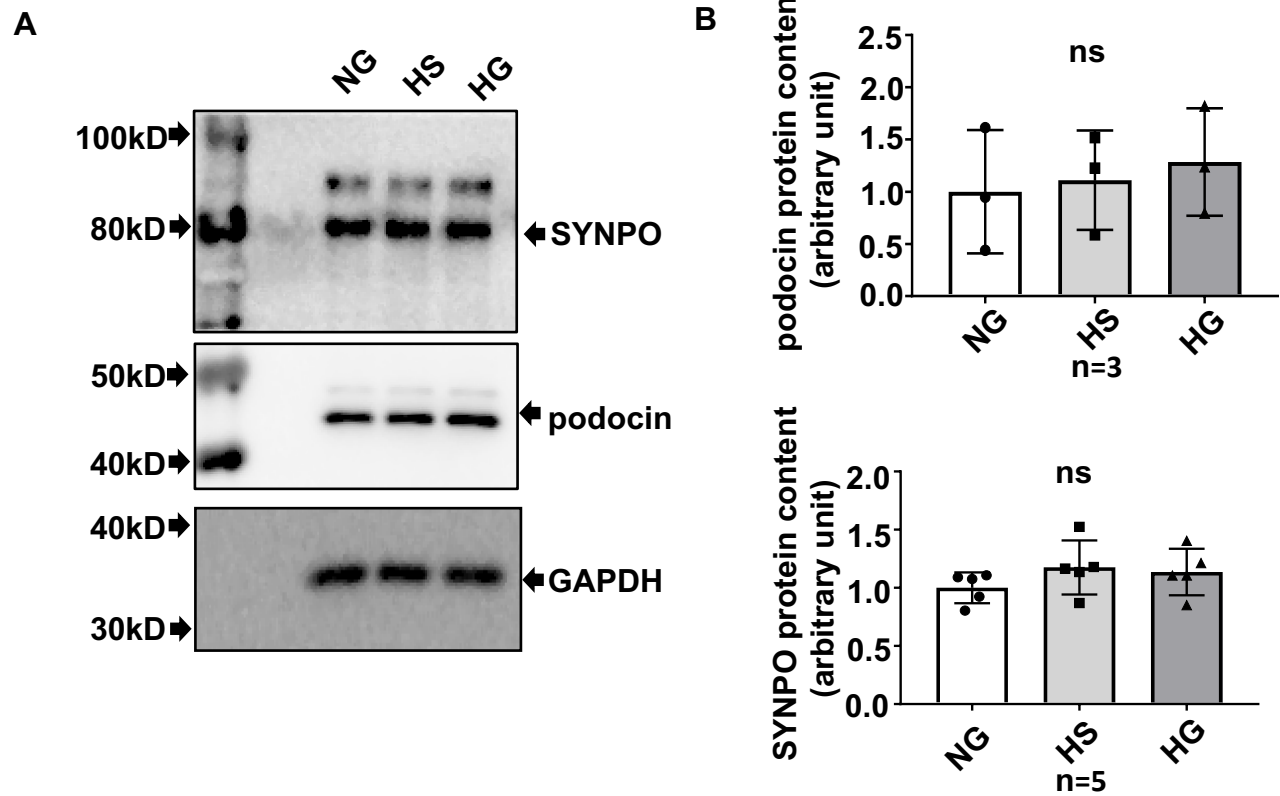

Supplement: Figure-S1-revision [file mmc1.pdf]
